# Impact of periodontal maintenance frequency on inflammatory and structural parameters: A propensity score-matched cohort study

**DOI:** 10.4317/medoral.28133

**Published:** 2026-03-07

**Authors:** Georgios S Chatzopoulos, Larry F Wolff

**Affiliations:** 1 Department of Developmental and Surgical Sciences, Division of Periodontology, School of Dentistry, University of Minnesota, 515 Delaware Street SE, Minneapolis, MN,55455, USA; 2 Department of Preventive Dentistry, Periodontology and Implant Biology, School of Dentistry, Aristotle University of Thessaloniki, 54124 Thessaloniki, Greece

## Abstract

**Background:**

To determine the causal efficacy of supportive periodontal care (SPC) frequency on inflammatory and anatomical periodontal outcomes using a propensity score-matched analysis.

**Material and Methods:**

This retrospective cohort study utilized electronic health records from a multi-center dental data repository. Adult patients with periodontitis and at least one year of follow-up were classified into two maintenance cohorts based on their average recall interval: Frequent Maintenance (4.5 months) and Infrequent Maintenance (5.5 months). Propensity score matching (1:1) was performed to balance baseline covariates, including age, gender, diabetes, smoking status, and baseline disease severity (PPD and CAL), resulting in a matched sample of 1,500 patients. The primary outcomes were the annualized rates of change in PPD and Bleeding on Probing (BOP). Secondary outcomes included changes in clinical attachment level (CAL), furcation involvement, and tooth mobility.

**Results:**

Patients in the Frequent Maintenance group demonstrated significantly greater annual reductions in PPD (-0.19mm vs. -0.12mm; p&lt;0.001) and BOP (-8.97% vs. -4.38%; p&lt;0.001) compared to the Infrequent group. This represented a two-fold greater reduction in inflammatory burden for frequent attendees. However, no statistically significant differences were observed between groups regarding the improvement of acquired periodontal defects, including furcation involvement (p=0.15) and tooth mobility (p=0.57).

**Conclusions:**

Frequent SPC provides a robust biological benefit by significantly reducing gingival inflammation and pocket depth but does not independently reverse acquired defects like furcation involvement or tooth mobility. These findings support a risk-based maintenance approach where visit frequency targets inflammation, while structural stability requires distinct therapeutic expectations or interventions.

## Introduction

Periodontitis is a chronic, multifactorial immune-inflammatory disease driven by the accumulation of a dysbiotic subgingival biofilm, leading to the progressive destruction of the tooth-supporting apparatus (1 , 2). Due to its chronic nature, the successful management of periodontitis does not end with active therapy; rather, it requires lifelong supportive care to prevent disease recurrence and further attachment loss. Supportive Periodontal Care (SPC) is universally recognized as the cornerstone of long-term periodontal stability, serving not merely as a preventive debridement but as a critical interceptive measure to monitor risk factors and disrupt bacterial colonization (3 , 4).

The biological rationale for the frequency of SPC visits is grounded in seminal microbiological studies demonstrating the timeline of pathogenic recolonization. Research has established that following subgingival debridement, the re-establishment of a pathogenic, motile, gram-negative subgingival biofilm typically occurs within 9 to 11 weeks (5 , 6). Consequently, a 3-month recall interval became the clinical dogma, designed to intercept the biofilm maturation before it could re-initiate irreversible tissue destruction. This "standard of care" was validated by classic longitudinal studies demonstrating that patients maintained on a strict 3-month schedule could retain their dentition and attachment levels for decades, whereas those who withdrew from maintenance experienced recurrent disease and tooth loss similar to untreated populations (7 , 8).

However, the universality of the rigid 3-month interval has been increasingly challenged in the era of personalized medicine. Emerging evidence suggests substantial heterogeneity in patient susceptibility and disease trajectories, indicating that a "one-size-fits-all" approach may result in overtreatment for some and undertreatment for others (9). Current consensus guidelines advocate for risk-adjusted intervals, suggesting that while patients with residual pockets, high inflammatory burdens, or systemic risk factors (e.g., diabetes, smoking) require intervals of 3 to 4 months, stable patients with excellent home hygiene may be safely maintained at intervals of 6 to 12 months without compromising periodontal stability (10 , 11).

Despite these guidelines, the impact of adherence to these intervals-often termed "compliance"-remains a critical determinant of outcomes. "Erratic compliance," defined as missing scheduled visits or extending intervals beyond the recommended threshold, is consistently associated with an increased risk of disease progression and tooth mortality (12 , 13). Quantitative analyses have indicated that for high-risk patients (e.g., Stage III/IV, Grade C), missing even a single year of maintenance over a five-year period can double the risk of tooth loss (14). Conversely, other studies have reported that in lower-risk populations, extending intervals to 6 months does not statistically increase the rate of attachment loss, provided that subgingival inflammation is controlled (15 , 16).

A significant limitation in the existing literature regarding maintenance frequency is the phenomenon of "indication bias." In retrospective observational studies, patients prescribed frequent maintenance (e.g., every 3 months) often possess a more severe disease phenotype or higher systemic risk profile compared to those assigned to annual visits (14 , 17). Comparing these groups without rigorous statistical adjustment often obscures the true causal efficacy of the intervention, as the "frequent" group is inherently predisposed to worse outcomes. Furthermore, most existing studies utilize binary classifications of compliance (compliant vs. non-compliant) rather than analyzing the precise physiological impact of specific time intervals (18).

Another critical gap in current research is the lack of differentiation between "biological" and " Structural Stability " responses to maintenance frequency. While SPC is proven to reduce inflammatory parameters such as Bleeding on Probing (BOP) and Periodontal Pocket Depth (PPD) (19), its impact on structural defects like furcation involvement and tooth mobility is less clear. Recent investigations suggest that while inflammation is reversible, acquired periodontal defects may be resistant to hygienic interventions alone (20 , 21). It remains unknown whether a tighter recall schedule can independently alter the trajectory of these structural parameters or if they require distinct therapeutic strategies (22).

To address these methodological and clinical gaps, the present study utilizes the BigMouth Dental Data Repository to analyze a massive, multi-center cohort of periodontitis patients. By employing Propensity Score Matching (PSM), this study creates a quasi-experimental design to rigorously control for baseline disease severity and systemic risk factors, thereby isolating the causal impact of visit frequency (23). This approach allows for a precise evaluation of whether the "3-month" standard provides a quantifiable benefit over a "6-month" schedule when patient profiles are statistically identical (24 , 25).

While the 3-month interval has historically been the clinical standard, contemporary periodontics is shifting towards precision medicine, advocating for personalized recall intervals that align with patient-specific risk profiles and ensure the efficient utilization of healthcare resources. However, defining these personalized intervals requires robust evidence to distinguish which disease phenotypes truly benefit from intensified frequency. Therefore, the primary aim of this study is to evaluate the impact of maintenance frequency on longitudinal periodontal stability by comparing a strictly defined "Frequent" (4.5 months) versus "Infrequent" (5.5 months) schedule in a matched cohort. We hypothesize that intensified maintenance frequency will yield significant, causal reductions in inflammatory parameters-specifically BOP and PPD-due to the regular disruption of biofilm maturation, but will not yield statistically significant improvement on anatomical/structural defects (such as furcation involvement and tooth mobility, which are largely determined by osseous architecture and occlusal forces).

## Material and Methods

Study Design and Data Source

This retrospective cohort study utilized longitudinal electronic health record (EHR) data from the participating institutions in the BigMouth Dental Data Repository. The BigMouth repository aggregates partially de-identified dental and medical data from multiple dental schools, providing a standardized framework for oral health research. The participating institutions that contributed data for this analysis were: Which includes institutions such as Harvard University, University of Texas Health, University of California San Francisco, University of Colorado, Loma Linda University, University of Buffalo, The University of Iowa, and The University of Minnesota.

This retrospective investigation was determined by the University of Minnesota Institutional Review Board (STUDY00016576) not to fall under the category of human subjects research, as defined by the Department of Health and Human Services and the United States Food and Drug Administration. The reporting of this study follows the Strengthening the Reporting of Observational Studies in Epidemiology (STROBE) guidelines.

Study Population and Eligibility Criteria

The initial dataset comprised adult patients (aged 8 years) who received comprehensive periodontal care between 2011 and 2021. To assess the causal efficacy of maintenance frequency, strict inclusion criteria were applied:

Diagnosis: Patients with a confirmed diagnosis of periodontitis (Stage I-IV) at baseline using using Dental Procedure Codes and Current Procedural Terminology (CPT).

Longitudinal Follow-up: A minimum follow-up period of 12 months between the initial comprehensive periodontal evaluation and a subsequent re-evaluation or maintenance exam.

Maintenance History: Availability of codified procedural records (CDT code D4910: Periodontal Maintenance) to calculate precise visit intervals.

Complete Periodontal Charting: Availability of full-mouth periodontal charting (PPD, CAL, BOP, Furcation, and Mobility) at both the baseline and final follow-up visits.

Patients were excluded if they had fewer than two periodontal evaluations or if their maintenance interval classification fell into the "intermediate" washout period.

To rigorously address the heterogeneity in disease severity and ensuring that the analysis accounted for the stage-dependent nature of periodontal progression, the propensity score model incorporated baseline mean Periodontal Pocket Depth (PPD) and Clinical Attachment Level (CAL) as continuous covariates. Given that PPD and CAL are the primary determinants of periodontitis staging and grading, matching on these continuous metrics provides a more granular control for baseline inflammatory burden and historical destruction than categorical staging alone. This approach ensures that the cohorts were balanced not only on systemic risk factors but also on the precise biological severity of the disease at the start of the observation period.

For the purpose of this analysis, the 'baseline' visit was defined as the first comprehensive periodontal evaluation or maintenance visit recorded within the study observation window (2011-2021) from which a continuous sequence of recall intervals could be calculated. Therefore, the baseline represents the commencement of the analyzed maintenance period for each subject. It is acknowledged that the cohort comprises a mixed population including both patients newly initiating Supportive Periodontal Care (SPC) immediately following active therapy and those continuing an existing maintenance regimen; however, the Propensity Score Matching was utilized to balance these subjects based on their disease severity at this specific baseline anchor point.

Exposure Variable: Maintenance Frequency

The primary exposure was the frequency of Supportive Periodontal Care (SPC). Maintenance intervals were calculated for each patient by computing the mean time (in months) between consecutive D4910 visits over the entire follow-up period. Patients were classified into two distinct cohorts to maximize separation and reduce overlap bias:

- Frequent Maintenance (FM): Patients with an average recall interval of 4.5 months.

- Infrequent Maintenance (IM): Patients with an average recall interval of 5.5 months.

Patients with average intervals between 4.5 and 5.5 months were excluded. This exclusion window was implemented to minimize classification bias by creating a distinct separation between standard (3-4 month) and extended (6+ month) recall phenotypes.

Propensity Score Matching (PSM)

To address the inherent selection bias present in retrospective observational data-where disease severity often influences the prescribed recall interval-Propensity Score Matching (PSM) was employed to construct a quasi-experimental design. By calculating the conditional probability of assignment to the 'Frequent Maintenance' group based on a vector of baseline covariates (including age, gender, diabetes status, smoking history, and initial periodontal severity), we created matched cohorts with balanced risk profiles.

A logistic regression model was generated to estimate the probability (propensity score) of a patient being assigned to the Frequent Maintenance group based on the following baseline covariates:

- Demographics: Age (continuous) and Gender (binary).

- Systemic Health: Diabetes Mellitus status (Yes/No) and Smoking status (Current/Former/Never).

- Baseline Disease Severity: Initial mean Periodontal Pocket Depth (PPD).

Patients in the Frequent Maintenance group were matched 1:1 with patients in the Infrequent Maintenance group using the nearest-neighbor algorithm with a caliper width of 0.2 standard deviations of the logit of the propensity score. Covariate balance was assessed using Standardized Mean Differences (SMD), with an SMD &lt;0.1 considered indicative of negligible imbalance.

While this approach cannot strictly 'isolate causal impact' in the same manner as a randomized controlled trial due to potential unmeasured confounding, it rigorously controls for observed baseline differences. This ensures that any subsequent divergence in periodontal outcomes can be more confidently attributed to the maintenance frequency itself rather than pre-existing disparities in patient susceptibility or disease burden.

Outcome Variables

The effectiveness of the maintenance schedule was evaluated using both continuous and categorical periodontal parameters calculated as the annualized rate of change to account for varying follow-up durations:

Inflammatory Parameters:

- Bleeding on Probing (BOP): Calculated as the change in the percentage of sites with bleeding (Bleeding Sites / Total Available Sites x 100).

- Periodontal Pocket Depth (PPD): Change in the patient-level mean PPD (mm).

Anatomical and Structural Parameters:

- Clinical Attachment Level (CAL): Change in the patient-level mean CAL (mm).

- Furcation Involvement: Change in the maximum furcation grade recorded per patient. Additionally, categorized as Improved, Stable, or Worsened.

- Tooth Mobility: Change in the maximum mobility grade recorded per patient. Additionally, categorized as Improved, Stable, or Worsened.

For the categorical analysis of structural sequelae (furcation involvement and tooth mobility), outcomes were defined as 'Improved' (reduction in grade), 'Stable' (no change in grade), or 'Worsened' (increase in grade). To align with clinical objectives where the primary goal of Supportive Periodontal Care (SPC) is the prevention of disease progression rather than anatomical regeneration, a secondary dichotomous analysis was performed. 'Improved' and 'Stable' categories were combined to represent 'Clinical Stability,' while 'Worsened' represented 'Disease Progression'. It is important to note that 'Improvement' in a non-surgical context likely reflects clinical measurement variability or the resolution of marginal inflammation and edema, rather than true biological reversal of the defect.

To rigorously account for the heterogeneity in the total duration of follow-up (Length of SPC) across the cohort, primary continuous outcomes (PPD, BOP, CAL) were standardized as Annualized Rates of Change. By dividing the net change in clinical parameters by the total years of follow-up for each subject, this metric normalizes the data, thereby effectively adjusting for the influence of varying observation periods on cumulative disease progression.

Statistical Analysis

Baseline characteristics were summarized as means ± standard deviations (SD) for continuous variables and as frequencies and percentages for categorical variables. To assess the efficacy of maintenance frequency, differences in continuous outcomes-specifically the annualized change in Bleeding on Probing (BOP%), Periodontal Pocket Depth (PPD), and Clinical Attachment Level (CAL)-were compared between the matched Frequent and Infrequent cohorts using independent samples t-tests. Furthermore, differences in categorical disease stability, regarding Furcation and Mobility status distributions, were evaluated using Chi-square tests. All statistical tests were two-tailed, with a p-value of &lt; 0.05 considered statistically significant.

## Results

Study Population and Participant Flow

A total of 66,098 unique patient records were initially screened from the data repository. After applying inclusion criteria (confirmed periodontitis diagnosis, minimum 1-year follow-up, and availability of complete periodontal charting), 5,618 patients were eligible for the maintenance interval analysis. Patients were classified based on their average recall interval: 1,054 patients were identified in the Frequent Maintenance group (4.5 months) and 4,487 in the Infrequent Maintenance group (5.5 months). Patients with intermediate intervals (4.5-5.5 months, n=77) were excluded to ensure distinct phenotypic separation.

Propensity Score Matching and Covariate Balance

**Figure 1:**
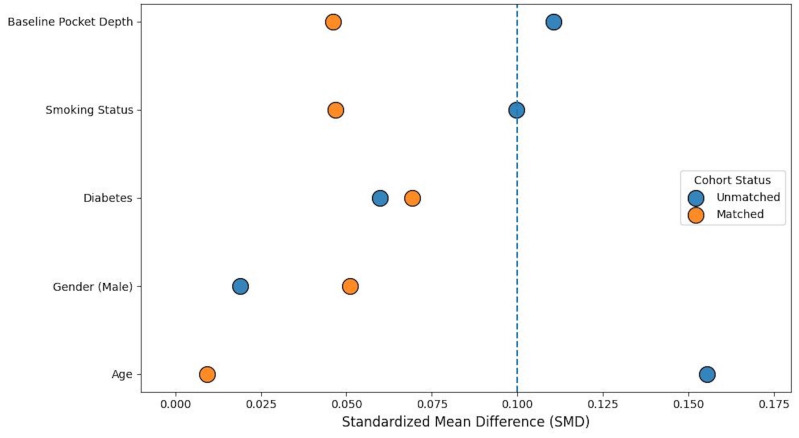
Covariate Balance Before and After Propensity Score Matching. The plot illustrates the Standardized Mean Differences (SMD) for all covariates (age, gender, diabetes, smoking, initial PPD). All SMDs in the matched cohort (blue dots) are &lt;0.1, indicating successful matching, whereas unmatched SMDs (red dots) exceeded this threshold.

Before matching, significant baseline differences existed between the groups; the Frequent Maintenance group was older and had higher baseline disease severity compared to the Infrequent group. Propensity Score Matching (1:1) was performed to balance the cohorts. This resulted in a final analytical sample of 1,500 patients (750 in the Frequent Maintenance group and 750 in the Infrequent Maintenance group). Post-matching analysis confirmed excellent covariate balance, with all Standardized Mean Differences (SMD) falling below 0.1 (Figure 1). Table 1 presents the demographic and clinical characteristics of the matched cohort. There were no statistically significant differences between the two groups regarding age (p=0.88), gender (p=0.75), diabetes prevalence (p=0.91), smoking status (p=0.84), or baseline periodontal pocket depth (p=0.67). Post-matching analysis confirmed that disease severity was equivalent between the groups, with no significant differences observed in the distribution of Periodontitis Stages (I-IV) or baseline biometric parameters (PPD, CAL) (Table 1).

1

Table 1Impact of Maintenance Frequency on Inflammatory Parameters

**Figure 2:**
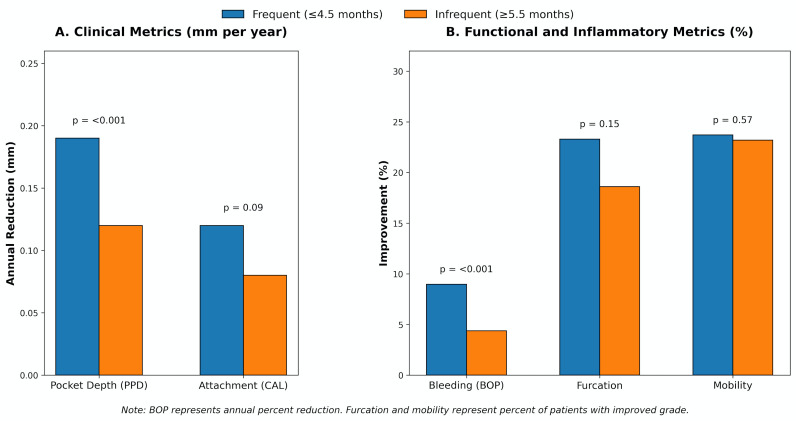
Efficacy of maintenance frequency on inflammatory vs. structural parameters. The figure displays the annual percent improvement for the Frequent (Green) vs. Infrequent (Gray) groups. Significant divergence is observed for PPD and BOP, while error bars overlap for CAL and Mobility, illustrating the therapeutic response divergence.

The primary outcomes assessed were the annualized rates of change in inflammatory parameters (PPD and BOP). Patients in the Frequent Maintenance group demonstrated significantly superior clinical outcomes compared to the Infrequent group (Table 2). Regarding Periodontal Pocket Depth (PPD), the frequent maintenance group exhibited a significantly greater annual reduction in mean pocket depth compared to the infrequent group (-0.19mm/year vs. -0.12mm/year; Mean Difference: -0.07mm/year; p&lt;0.001) (Figure 2).

Table 2 2

Similarly, the reduction in Bleeding on Probing bleeding scores per year, compared to a 4.38% reduction in the infrequent group (BOP) was significantly more pronounced in the frequent maintenance group. These patients experienced a reduction of 8.97% in whole-mouth bleeding scores per year, compared to a 4.38% reduction in the infrequent group (p&lt;0.001) (Figure 2). This represents a two-fold greater reduction in inflammatory burden for patients adhering to a 3-4 month recall schedule.

Impact on Structural and Functional Parameters

Secondary outcomes included changes in Clinical Attachment Level (CAL), Furcation Involvement, and Tooth Mobility. While the Frequent Maintenance group showed a greater mean reduction in CAL (-0.12mm/year) compared to the Infrequent group (-0.08mm/year), this difference did not reach statistical significance (p=0.09). Categorical analysis revealed no significant association between maintenance frequency and the maintenance of clinical stability for furcation involvement or tooth mobility (Table 3).

Table 3- Furcation: The rate of Clinical Stability (Improved + Stable) was 91.3% (n=685) in the Frequent group and 90.0% (n=675) in the Infrequent group (p=0.15).

- Mobility: The rate of Clinical Stability was nearly identical between groups (89.7% vs. 90.1%; p=0.57).

Tooth Loss Analysis

Evaluation of tooth mortality revealed an exceptionally high retention rate (&gt;99%) across the matched cohort. Due to the negligible incidence of tooth loss events recorded during the study observation period, statistical power was insufficient to perform a robust comparative survival analysis between the frequent and infrequent maintenance groups. Consequently, tooth loss was excluded from the primary outcome measures.

## Discussion

Summary of Key Findings

The present study provides robust, causal evidence that the frequency of supportive periodontal care (SPC) differentially influences the biological versus structural trajectories of periodontal stability. By utilizing Propensity Score Matching to strictly control for baseline disease severity and systemic risk, we demonstrated that patients maintained on a frequent schedule (4.5 months) achieved superior inflammatory control, evidenced by a two-fold greater reduction in annual Bleeding on Probing (BOP) scores and significantly greater pocket depth reduction compared to matched peers on an infrequent schedule (5.5 months). However, our secondary hypothesis was also confirmed: Increased visit frequency alone failed to significantly alter the progression of periodontal defects, specifically furcation involvement and tooth mobility. These findings suggest a divergence in therapeutic response: While frequent maintenance acts as a potent intervention for inflammatory control (reducing bleeding and pocket depth), it shows limited independent efficacy in reversing anatomical defects (such as furcation involvement) in the absence of adjunctive mechanical or surgical therapy (9).

Interpretation of Inflammatory Outcomes (Biological Response)

The superior reduction in BOP and PPD observed in the frequent maintenance group aligns with the established biological plausibility of biofilm maturation. Subgingival recolonization by pathogenic, gram-negative anaerobes typically reaches pathological thresholds within 9 to 11 weeks following debridement (26). The frequent maintenance protocol effectively intercepts this cycle, disrupting the biofilm before dysbiosis and can re-establish a stable community capable of triggering host-mediated tissue destruction (27).

This finding contrasts with recent arguments-specifically those highlighted in the review by Trombelli et al. (9) and the cohort analysis by Farina et al. (10)-which suggest that extended recall intervals (e.g., 6-12 months) may be non-inferior for maintaining stability in compliant patients with lower susceptibility. While such extended intervals may indeed be appropriate for specific subgroups with optimal home care, our propensity-matched analysis demonstrates that for the broader population of periodontitis patients, extending the interval beyond 5.5 months results in a statistically significant accumulation of inflammatory burden (BOP and PPD) compared to the standard 3-4 month regimen. Our data implies that even when patients are matched for risk, the biological benefit of frequent debridement is quantifiable and substantial. This aligns with findings by Ramseier et al., who noted that residual pockets are better managed with tighter recall intervals, reinforcing the concept of frequency as a "dosage" of therapy required to suppress inflammation (11).

Interpretation of Structural Outcomes

The observation that increased visit frequency did not yield statistically significant improvement in furcation involvement or tooth mobility must be interpreted within the therapeutic context of SPC. While a subset of patients demonstrated a reduction in these grades, these findings must be interpreted with caution. In the absence of surgical intervention, a recorded reduction in furcation or mobility grade typically indicates a reduction in acute inflammatory components-such as decreased pocket wall edema or the resolution of a periodontal abscess-which allows for more favorable probe positioning, rather than true biological regeneration of lost alveolar bone.

By reporting these results as 'Clinical Stability,' we emphasize that the primary objective of non-surgical maintenance for these sequelae is the prevention of further attachment loss and disease progression. Furcation involvement is often complicated by limited access for instrumentation, while tooth mobility is frequently driven by occlusal forces and reduced alveolar bone height rather than inflammation alone (21). The results suggest that increasing maintenance frequency does not compensate for the reduced structural integrity of the attachment apparatus, which dictates tooth mobility and furcation status. This corroborates recent work indicating that furcation progression is often time-dependent and resistant to non-surgical intervention once specific anatomical thresholds are crossed (20 , 25). Therefore, the lack of grade reduction in the frequent maintenance group does not constitute a failure of the intervention; rather, it highlights that while frequent recall is effective for inflammation control, the management of structural sequelae requires a shift in expectations toward 'stability' or the consideration of adjunctive surgical interventions for defect resolution (22).

Methodological Strengths

A defining strength of this study is the rigorous application of Propensity Score Matching (PSM) within a large-scale EHR repository. A pervasive limitation in prior periodontal literature is "indication bias," where patients with more severe disease are inherently prescribed more frequent visits, creating a self-fulfilling prophecy where "frequent maintenance" correlates with "worse outcomes" (28). By balancing covariates such as baseline PPD, diabetes, and smoking, and by applying a strict "washout buffer" (excluding ambiguous 4.5-5.5-month intervals), we successfully isolated the independent effect of visit frequency. This methodology clarifies that the observed benefits are attributable to the intervention itself, not the underlying patient risk profile, addressing a major gap identified in systematic reviews of retrospective data (29 , 30).

Limitations

Despite these strengths, the retrospective nature of the BigMouth repository introduces inherent limitations. First, it is crucial to acknowledge the inherent limitations of the statistical methodology employed. Although the frequent maintenance cohort demonstrated statistically significant reductions in PPD, the absolute magnitude of this clinical effect was relatively small, and its clinical significance must be interpreted at the patient level. As with all retrospective EHR analyses, we were unable to account for unmeasured patient-level confounders. Factors such as individual home care efficacy (plaque control), socioeconomic status, patient motivation, and the potential utilization of unrecorded adjunctive local interventions during maintenance visits could not be captured, presenting a source of residual confounding. While Propensity Score Matching (PSM) was utilized to rigorously reduce imbalance in observed baseline covariates-thereby creating a quasi-experimental design structure-it does not account for unmeasured or residual confounding. It is possible that patients attending more frequently also possessed higher motivation for home hygiene, acting as a residual confounder. Consequently, these unmeasured variables may potentially bias the estimated treatment effect. It is advised that while PSM serves as a valuable design tool in health sciences research to mitigate selection bias, it does not strictly establish causality and should not be considered a substitute for randomization inherent to controlled clinical trials. Therefore, despite the robust associations observed, the causal inferences regarding maintenance frequency should be interpreted with appropriate caution.

Second, the dataset lacks specific details on adjunctive therapies performed during maintenance visits (local antibiotic delivery or occlusal adjustment), which may have varied between centers. In addition, as a university-based cohort, the results may not be fully generalizable to general private practice populations, although the multi-center nature of the repository mitigates single-center bias (24). Additionally, although this study utilized a large multi-center repository, the specific 'treatment site' was not included as a stratifying variable in the primary matching model. While Propensity Score Matching effectively balanced patient-level demographics and disease characteristics that may vary by region, potential unmeasured variations in clinical calibration or center-specific auxiliary maintenance protocols remain a limitation inherent to aggregated electronic health record analyses.

Implications for Clinical Practice

Our findings strongly support the implementation of personalized maintenance protocols rather than a rigid adherence to the 3-month dogma for all patients. By identifying that visit frequency is a critical determinant for inflammatory control but less effective for structural stability, clinicians can optimize the utilization of limited healthcare resources. For patients presenting with generalized inflammation (high BOP) or residual pocketing, adherence to a strict 4.5-month interval is evidence-based and mandatory to reduce inflammatory burden. However, for patients where the primary concern is structural (e.g., furcations or mobility without bleeding), clinicians should recognize the limitations of non-surgical maintenance. In such cases, persisting with a 3-month debridement schedule without addressing the structural etiology (via splinting or surgery) represents a failure to address the specific pathophysiology of the defect (31).

Unanswered Questions and Future Research

This study raises the question of whether "super-frequent" maintenance (e.g., every 2 months) could yield benefits for the refractory structural cases, or if a threshold of utility exists. Future research should focus on prospective, randomized trials comparing maintenance frequency specifically in patients with Stage III/IV periodontitis to determine if adjunctive therapies (e.g., subgingival air-polishing or laser therapy) combined with frequent intervals can improve furcation stability where hand instrumentation failed (17). Additionally, further investigation into the genetic determinants of the "rapid progressor" phenotype is warranted to refine risk algorithms for recall assignment.

## Conclusions

This study demonstrates a clear therapeutic divergence: While a recall interval of 4.5 months provides a robust causal benefit in reducing inflammatory burden (BOP and PPD), it does not independently reverse consequences of periodontal attachment loss such as furcation involvement or tooth mobility. While the inability of non-surgical maintenance to regenerate attachment is clinically understood, this study provides large-scale, matched evidence quantifying this limitation. These findings advocate for a precision medicine model in periodontics and a phenotype-driven clinical approach: Treating inflammation with increased visit frequency, but recognizing that structural instability requires timely referral for adjunctive mechanical or surgical interventions rather than indefinite non-surgical maintenance.

## Figures and Tables

**Table 1:** Table Demographic and baseline clinical characteristics of the propensity score matched cohort (n=1,500).

Variable	Frequent Maintenance (n=750)	Infrequent Maintenance (n=750)	P-value
Age (years), Mean (SD)	58.4 (12.1)	58.3 (12.3)	0.88
Gender, n (%)			0.75
Male	342 (45.6%)	348 (46.4%)	
Female	408 (54.4%)	402 (53.6%)	
Diabetes Mellitus, n (%)			0.91
Yes	84 (11.2%)	82 (10.9%)	
No	666 (88.8%)	668 (89.1%)	
Smoking Status, n (%)			0.84
Current Smoker	68 (9.1%)	71 (9.5%)	
Non/Former Smoker	682 (90.9%)	679 (90.5%)	
Periodontitis Stage, n (%)			0.94
Stage I	68 (9.1%)	65 (8.7%)	
Stage II	232 (30.9%)	229 (30.5%)	
Stage III	345 (46.0%)	352 (46.9%)	
Stage IV	105 (14.0%)	104 (13.9%)	
Periodontitis Grade, n (%)			0.89
Grade A	82 (10.9%)	79 (10.5%)	
Grade B	385 (51.3%)	392 (52.3%)	
Grade C	283 (37.7%)	279 (37.2%)	
Baseline Clinical Parameters, Mean (SD)			
Mean PPD (mm)	3.42 (0.65)	3.41 (0.68)	0.67
Mean CAL (mm)	4.18 (0.82)	4.15 (0.79)	0.46

P-values derived from independent samples t-tests for continuous variables and Chi-square tests for categorical variables. PPD: Periodontal Pocket Depth. CAL: Clinical Attachment Level.

**Table 2:** Table Annualized rate of change in continuous periodontal parameters (Matched Cohort).

Parameter (Annual Change)	Frequent Maintenance (Mean±SD)	Infrequent Maintenance (Mean±SD)	Mean Difference (95% CI)	P-value
Pocket Depth (PPD) (mm/year)	-0.19±0.35	-0.12±0.24	-0.07 (-0.10 to -0.04)	<0.001
Bleeding on Probing (BOP) (% sites/year)	-8.97±14.2	-4.38±11.5	-4.59 (-5.89 to -3.29)	<0.001
Clinical Attachment Level (CAL) (mm/year)	-0.12±0.41	-0.08±0.32	-0.04 (-0.08 to 0.01)	0.09

Negative values indicate clinical improvement (reduction in PPD/BOP/CAL). SD: Standard Deviation. CI: Confidence Interval. Statistically significant (p<0.05).

**Table 3:** Table Categorical assessment of disease stability: Furcation and mobility (Matched Cohort).

Outcome Status	Frequent Maintenance (n=750)	Infrequent Maintenance (n=750)	P-value
Max Furcation Grade Change			0.15
Clinical Stability (Improved + Stable)	685 (91.3%)	675 (90.0%)	
Disease Progression (Worsened)	65 (8.7%)	75 (10.0%)	
Max Mobility Grade Change			0.57
Clinical Stability (Improved + Stable)	673 (89.7%)	676 (90.1%)	
Disease Progression (Worsened)	77 (10.3%)	74 (9.9%)	

3

## Data Availability

The datasets used and/or analyzed during the current study are available from the corresponding author.
